# Fighting biofilms with lantibiotics and other groups of bacteriocins

**DOI:** 10.1038/s41522-018-0053-6

**Published:** 2018-04-19

**Authors:** Harsh Mathur, Des Field, Mary C. Rea, Paul D. Cotter, Colin Hill, R. Paul Ross

**Affiliations:** 1 Teagasc Food Research Centre, Moorepark, Fermoy, County Cork Ireland; 2 APC Microbiome Ireland, University College Cork, Cork, Ireland; 3 School of Microbiology, University College Cork, Cork, Ireland; 4 College of Science, Engineering and Food Science, University College Cork, Cork, Ireland

## Abstract

Biofilms are sessile communities of bacteria typically embedded in an extracellular polymeric matrix. Bacterial cells embedded in biofilms are inherently recalcitrant to antimicrobials, compared to cells existing in a planktonic state, and are notoriously difficult to eradicate once formed. Avenues to tackle biofilms thus far have largely focussed on attempting to disrupt the initial stages of biofilm formation, including adhesion and maturation of the biofilm. Such an approach is advantageous as the concentrations required to inhibit formation of biofilms are generally much lower than removing a fully established biofilm. The crisis of antibiotic resistance in clinical settings worldwide has been further exacerbated by the ability of certain pathogenic bacteria to form biofilms. Perhaps the most notorious biofilm formers described from a clinical viewpoint have been methicillin-resistant *Staphylococcus aureus* (MRSA), *Staphylococcus epidermidis*, *Pseudomonas aeruginosa*, *Gardnerella vaginalis* and *Streptococcus mutans*, the latter of which is found in oral biofilms. Due to the dearth of novel antibiotics in recent decades, compounded by the increasing rate of emergence of resistance amongst pathogens with a propensity for biofilm formation, solutions are urgently required to mitigate these crises. Bacteriocins are a class of antimicrobial peptides, which are ribosomally synthesised and often are more potent than their antibiotic counterparts. Here, we review a selection of studies conducted with bacteriocins with the ultimate objective of inhibiting biofilms. Overall, a deeper understanding of the precise means by which a biofilm forms on a substrate as well as insights into the mechanisms by which bacteriocins inhibit biofilms is warranted.

## Introduction

Biofilms are composed of cells in a sessile state found in a polymeric matrix, and can be attached to both biotic and abiotic substrates.^1–4^ Biofilms have been known to contribute to physical and chemical protection as well as protection from antimicrobials.^2^ In clinical settings, the survival of biofilms on medical devices and hospital equipment permits certain pathogens to easily infect patients. Once infected, pathogen-associated biofilms can evade human host immune defences and are frequently associated with persistent infections, often resistant to antibiotic therapy.^5^ The stages involved in the formation of a biofilm are quite complex and several comprehensive reviews have already described the biofilm growth cycle, whereby bacteria adhere to a substrate, followed by maturation of the biofilm and subsequent release of clusters of cells from the matrix of the biofilm (see reviews by Garrett et al. and Flemming & Wingender).^6,7^ The concentrations of antibiotics needed to eradicate a biofilm can often range from 100 to 1000× minimum inhibitory concentration (MIC) of that needed to kill planktonic cells.^5,8,9^ Aside from antimicrobial resistance, bacteria present in a biofilm are also resistant to various physicochemical stresses, enabling biofilms to persist in even the harshest of conditions. In the food industry, biofilms have the ability to cause food-borne disease outbreaks. Furthermore, inefficient cleaning regimes may be a contributing factor in the spread of resistance in hospital environments^10^. Thus, there has been a growing emphasis on attempting to prevent the initial stages of biofilm formation, in lieu of targeting fully formed biofilms.^11^ Thus far, attempts have been made to target adhesion, quorum sensing and dispersion of biofilms, each of which are critical steps in the formation of a fully established biofilm.^12^ A recent comprehensive review described the efficacy of antibiotics in combination with other antimicrobial peptides and essential oils, as well as the effectiveness of biofilm-degrading enzymes, quorum sensing inhibitors and nano particles as potential anti-biofilm agents.^13^

Due to the widespread resistance of biofilms to conventional antibiotics, one alternative avenue to tackle this problem is to harness bacteriocins as antimicrobials either independently or in combination with existing proven antimicrobials, with a view to targeting biofilms. Bacteriocins are antimicrobial peptides produced by bacteria typically 2–10 kDa in size, generally targeting closely related bacteria, and are ribosomally synthesised in nature.^14,15^ They are broadly classified into two main groups, namely class I (post-translationally modified) and class II (unmodified) bacteriocins. By far the most extensively studied subgroup is the lantibiotics belonging to class I bacteriocins. These are characterised by the unusual amino acids, dehydroalanine and dehydrobutyrine, as a result of the dehydration of serine and threonine residues, as well as the presence of lanthionine (Lan) and β-methyllanthionine (MeLan) intramolecular bridges.^16,17^ Unsurprisingly, the vast majority of bacteriocins which have been studied thus far with a view to targeting biofilms are lantibiotics. The various applications of bacteriocins, including food preservation, health benefits, as well as anti-biofilm activity were already addressed in a recent review.^18^

Critically, unlike antibiotics, the ribosomally-synthesised nature of bacteriocins renders them one of the most amenable agents to manipulate or bioengineer to target specific pathogens and biofilm formers. Indeed, several studies have reported enhanced bioactivity and/or physicochemical properties of bacteriocins including ameliorated diffusion properties and stability in different pH conditions, traits which have been strategically bioengineered.^19–27^ Perhaps the most thoroughly studied bacteriocin has been the lantibiotic nisin. This lantibiotic has already been reported to be effective at permeating biofilms and the opportunity exists to improve this characteristic by seeking derivatives which have an enhanced ability to diffuse through the various complex strata in a biofilm.^28–31^ Such increased diffusion properties would not only make them more potent against biofilms but also effective in various food systems, targeting potential food-borne biofilm formers such as *Listeria monocytogenes*. In addition, the advantages of generating bioengineered derivatives of bacteriocins with augmented bioactivity can also reduce costs associated with targeting biofilms, as lower concentrations of the relevant peptide(s) will be needed, relative to the wild-type parental peptide(s).^32^ Furthermore, as the majority of bacteriocins are marginally cationic in nature, they have a tendency to be naturally attracted to anionic surfaces. Impregnation of such anionic surfaces with these cationic bacteriocins can also potentially render such peptides useful against biofilms forming on such a surface. In addition, bacteriocins, and especially those produced by lactic acid bacteria generally exhibit relatively low levels of cytotoxicity towards human and animal tissues. Indeed, the non-toxic nature of nisin has been highlighted on a number of occasions,^33,34^ while Murinda et al. also reported that the bacteriocins nisin, pediocin and colicin E6 displayed little or no cytotoxicity towards Vero Monkey Kidney cells.^35^ Rare exceptions do exist however, such as the *Enterococcus*-associated cytolysin, which has demonstrated cytotoxicity.^36^

In this review, we summarise the findings from some key recent studies which have utilised bacteriocins as a means to target biofilms. Due to their potency and efficacy against biofilms which is comparable to conventional antibiotics, bacteriocins could be harnessed as alternative and/or adjunctive therapeutic options to combat biofilms. The use of bacteriocins as adjuncts to antibiotics also has the potential to somewhat curtail the widespread problem of antibiotic resistance.

## Lantibiotics against biofilms

The biological value of several bacteriocins is further enhanced by their activity against biofilms, which are notoriously challenging to eradicate using conventional antibiotics. The lantibiotics are by far the most extensively studied subclass of bacteriocins and thus it is no surprise that the majority of studies with bacteriocins targeting biofilms have predominantly involved this group. Several studies have also explored combinations of lantibiotics with other bacteriocins or stressors with a view to either preventing the formation of biofilms and/or eradicating existing biofilms. In one such study, Mataraci and Dosler investigated the potency of several antibiotics combined with nisin against MRSA ATCC43300 planktonic cells and biofilms.^12^ *S. aureus* is a pathogen causing several skin infections as well as other systemic infections and several authors have already described the importance of biofilms in its pathogenicity.^37–44^ Synergistic interactions were found with antibiotic–nisin combinations, in terms of fractional inhibitory concentration determinations, whereas additive effects were obtained against planktonic cells of MRSA in the study by Mataraci and Dosler. Furthermore, antibiotic–nisin combinations were effective at preventing biofilm formation at 1× MIC.^12^ In contrast, however, biofilm-associated bacteria were highly resistant to antibiotics or nisin–antibiotic combinations. In a follow-up study by the same group, it was determined using time-kill assays that nisin acted synergistically with the antibiotics ciprofloxacin/daptomycin against MRSA biofilms.^45^ Okuda and co-workers also assessed the antimicrobial potencies of the lantibiotics nisin and nukacin ISK-1, as well as the bacteriocin lacticin Q against biofilms of MRSA.^46^ Nukacin ISK-1 is a class II lantibiotic^47,48^ while lacticin Q is a broad spectrum unmodified bacteriocin and thus has been categorised into a new family of class II bacteriocins.^49,50^ It was shown that while the glycopeptide antibiotic vancomycin was ineffective against MRSA biofilms, lacticin Q and nisin exhibited bactericidal activity against the biofilm, with nisin displaying more potent activity compared to lacticin Q.^46^ While the lantibiotic nukacin ISK-1 failed to inhibit MRSA biofilms, it did possess strong activity against planktonic cells. Overall, the study unveiled that the bacteriocins elicited pore formation and a consequent efflux of ATP from the biofilms and that this is an important mechanism of action in targeting MRSA biofilms.^46^

Sub-lethal concentrations of the lantibiotic nisin and the class II bacteriocin bovicin HC5 have also been shown to disrupt *S. aureus* adhering to polystyrene.^51^ Adhesion to abiotic surfaces such as polystyrene is a key step in the formation of biofilms. Interestingly, in the study by Pimentel-Filho et al. application of nisin and bovicin rendered the surfaces more hydrophilic and alterations in the free energy of adhesion between the polystyrene surfaces and bacterial cells prevented adhesion to the surface.^51^ Thus, modifications in the hydrophobicity of abiotic surfaces as well as bacterial cell surfaces triggered by bacteriocins can hinder this critical adhesion stage in biofilm formation. Importantly, it was determined that nisin and bovicin also had an impact on the transcription of certain genes in *S. aureus*, primarily affecting *clfB*, *fmnbA* and *icaD*, which are involved in biofilm formation. In another study, the impact of lysozyme and nisin on the biofilm forming ability of 25 *S. aureus* strains was evaluated and the authors found that the presence of nisin at 1× MIC inhibited the formation of biofilms, while sub-lethal concentrations failed to have any inhibitory effect.^52^

**Fig. 1:**
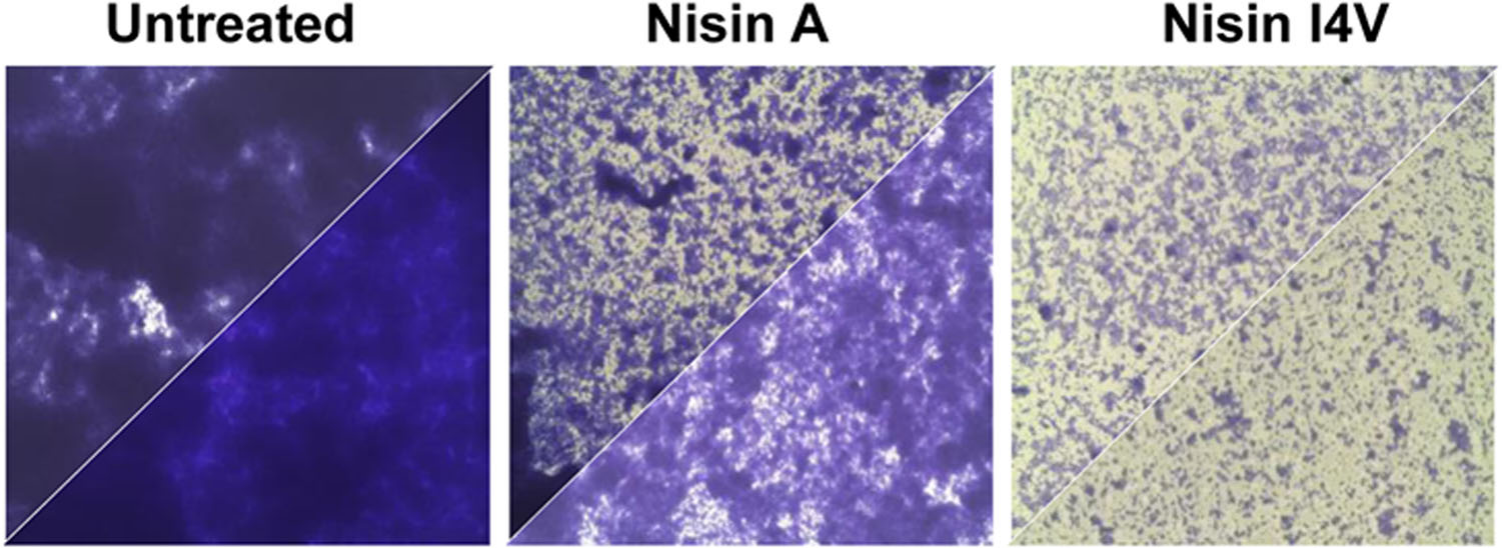
Biofilms treated with nisin assessed by microscopy: Assessment of *S. pseudintermedius* DK729 (top triangle) and *S. pseudintermedius* DSM21284 (bottom triangle) biofilms (magnification 1000×) after 24 h treatment with 16× MIC of nisin A (Wt) and nisin I4V peptides. (Adapted from Field et al. 2015c ^31^ under the terms of the Creative Commons Attribution License)

With regards to other *Staphylococcus* biofilm formers, one study demonstrated the efficacy of the lantibiotic gallidermin against *S. epidermidis* as well as against *S. aureus* biofilms.^53^ *S. epidermidis* exists as a commensal organism on the skin but has also been implicated in causing several nosocomial infections.^54^ Indeed, *S. epidermidis* has been described as the most common causative agent of medical device-associated infections and its ability to form biofilms is a key contributing factor in its pathogenic potential.^55^ Encouragingly, gallidermin was shown to prevent the growth of the *Staphylococcus* strains as well as inhibiting the formation of biofilms.^53^ However, the killing effect of gallidermin was diminished against 1 day-old and 5 day-old biofilms. Worryingly, approximately 0.1 to 1.0% of cells exposed to the lantibiotic were persister cells which survived gallidermin treatment.^53^ Persister cells are cells which reside in a dormant state in microbial communities and typically exhibit resistance to antimicrobials. Indeed, it has been suggested that the presence of persister cells may be the predominant reason for chronic infections displaying antibiotic resistance.^56,57^ Such persister cells may also be an important part of biofilms and likely contribute to the antibiotic-resistant properties of biofilms.^58,59^. With regards to *S. epidermidis* biofilms, a study by Davison et al. demonstrated that 50 µg/ml nisin was able to permeate through the biofilm cell clusters in 4 min, when assessed by using continuous flow models and confocal laser scanning microscopy.^28^ Unlike other antimicrobials used in the study, the lantibiotic elicited a quick and steady loss of green fluorescence from the biofilm, suggesting that permeation was highly effective across all the strata of the biofilm. During transit through the complex matrix of the biofilm, the lantibiotic was able to cause a loss of cell membrane integrity and consequently elicit a loss of viability of cells within the biofilm. Significantly, penetration of nisin through biofilms and planktonic cells occurred at a largely similar rate. It was also reassuring that a subpopulation of cells exhibiting decreased sensitivity to the lantibiotic was not seen. Overall, the authors concluded that nisin was effective at killing the cells in the biofilm but not effective at removing the existing biofilm.^28^ Recent studies have also reported the activity of nisin and bioengineered variants thereof against other *Staphylococcus* biofilms. Indeed, the nisin variant I4V was particularly effective at inhibiting the formation of *Staphylococcus pseudintermedius* DSM21284 biofilms and decreasing the biomass of established biofilms (Fig. 1).^31,32^ This canine pathogen is implicated in skin and wound infections and resistance to the antibiotic methicillin is common. Significantly, one study evaluated the ability of *S. pseudintermedius* isolated from dogs in forming biofilms and the study reported that a staggering 96% of the strains assessed were able to form biofilms.^60^ As expected, there was no correlation found between methicillin sensitivity or resistance and the ability to form biofilms.^60^

Food-borne pathogens have also been associated with the formation of biofilms, permitting them to persist in a viable state in food production environments.^61,62^ Although primarily found in a planktonic state, *L. monocytogenes* also has the ability to form biofilms under certain conditions.^63–67^ Consequently, several groups have evaluated the efficacy of lantibiotics against biofilms of *L. monocytogenes*. In one such study, the efficacy of nisin in combination with the essential oil cinnamaldehyde, and the food additive citric acid, at targeting biofilms of *L. monocytogenes* strain F2635 was reported. More specifically, 0.1 μg/ml of the bioengineered nisin derivative, M21A, independently and in combination with 175 μg/ml citric acid or 35 μg/ml cinnamaldehyde was particularly potent against F2635 biofilms, more so than combinations involving wild-type nisin A.^68^ In a separate study, the resistance properties of 4 and 11 day-old *L. monocytogenes* biofilms were assessed by measuring lethal dose 90 values of nisin, as well as assessment of biofilms via microscopy.^69^ Interestingly, the authors described that *L. monocytogenes* strain 4032 which formed biofilms on both polypropylene and stainless steel (SS) assumed a ‘cloud-type’ structure which was thought to contribute to resistance to nisin as well as other biocides. Importantly however, such resistance was associated with mature biofilms and not early stage biofilms or planktonic cells.^69^ The anti-biofilm activities of several bacteriocins including the two-peptide lantibiotic lichenicidin and the single peptide lantibiotics nisin Z and subtilomycin, were assessed in a recent study, showing that the bacteriocins were effective at inhibiting formation of *L. monocytogenes* biofilms and decreasing the viability of biofilms already formed.^70^ A separate study demonstrated that 4000 IU/ml nisin elicited a 57% reduction in *L. monocytogenes* biofilm formation, an 87% reduction in *Salmonella enteriditis* and a 30% decrease in *S. aureus* biofilm formation.^71^ The efficacy of high hydrostatic pressure (HHP) in combination with nisin in low pH conditions on targeting *L. monocytogenes* biofilms has also been evaluated.^72^ Inactivation of cells in the *L. monocytogenes* biofilm was apparent subsequent to HHP treatment when cells were treated in tryptic soy broth (TSB) pH5 supplemented with nisin. Furthermore, it was found that the presence of nisin in TSB at pH5 elicited a 5-log cfu/ml decrease in cell numbers and the cells became shorter in size.^72^

**Fig. 2:**
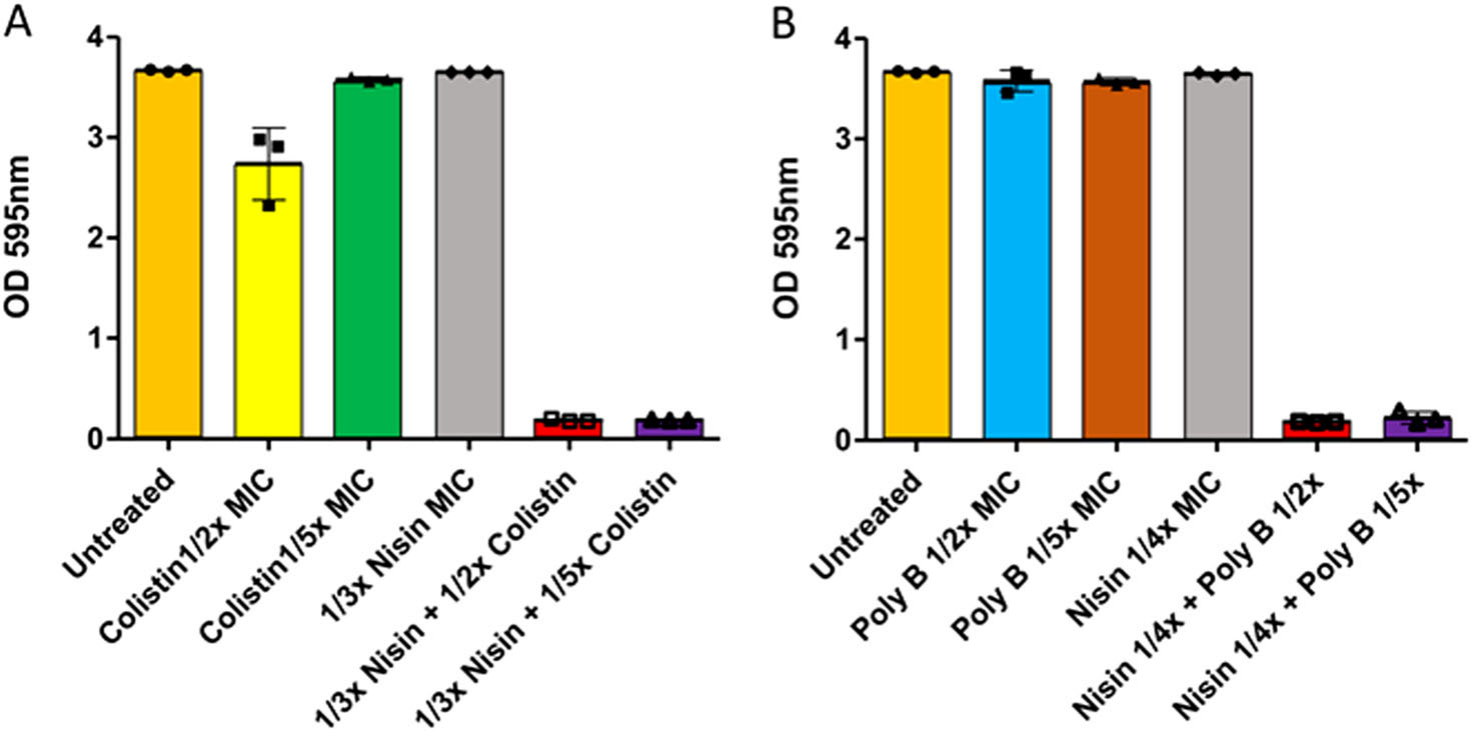
Anti-biofilm activity of nisin and polymyxins against *P. aeruginosa*: Inhibition of biofilm formation of *P. aeruginosa* PA-01 **a**) in the presence of nisin (1/3× MIC), colistin (1/2×, 1/5× MIC) and combinations thereof and **b**) in the presence of nisin (1/4× MIC) and polymyxin B (1/2×, 1/5× MIC) and combinations thereof, when assessed in microtiter plates and subjected to crystal violet (CV) staining for the detection of biofilm formation. (Adapted from Field et al. 2016b ^73^ under the terms of the Creative Commons Attribution License)

Studies investigating the potency of lantibiotics with a view to targeting biofilms formed by Gram negatives have also been explored. For instance, a recent study reported that nisin-polymyxin combinations were effective at inhibiting the formation of *Pseudomonas aeruginosa* biofilms (Fig. 2).^73^ *P. aeruginosa* is classified as an opportunistic Gram negative pathogen and is known to colonise and form biofilms in the lungs of cystic fibrosis patients.^8,74,75^ Field and co-workers demonstrated that the dose of polymyxin needed to prevent formation of *P. aeruginosa* biofilms was reduced in the presence of nisin. Nisin and related lantibiotics in general exhibit poor activity against Gram negative bacteria, primarily due to the presence of the outer membrane, which prevents nisin accessing its target, lipid II, in the cytoplasmic membrane.^76–80^ It is plausible that by binding the lipid A component of lipopolysaccharide, and thereby permeabilizing the outer membrane of *P. aeruginosa*, the transit of nisin to its target lipid II in the cytoplasmic membrane is facilitated by polymyxin, resulting in the apparent synergistic effect observed by Field et al.^73^. Indeed, previous studies had also demonstrated the synergistic effects of nisin in combination with polymyxins against the Gram negatives *P. aeruginosa* and *E. coli*. ^81–83^ A synergistic interaction of this nature could be a potential avenue to curtail the nephrotoxicity associated with polymyxins when treating *P. aeruginosa* infections clinically.

## Lantibiotics against oral biofilms

Perhaps the most common biofilm found in the human body is dental plaque in the oral cavity and is associated with the causation of dental caries and periodontal disease.^84–88^ Thus, several investigators have examined various means to minimise the negative effects associated with dental plaque biofilms. In particular, *Strep. mutans* is a key organism implicated in the formation of dental biofilms and is the primary causative agent of caries.^89^ In contrast, oral biofilms containing *Streptococcus sanguinis* may be associated with good oral health as periodontal disease has been linked with decreased *Strep. sanguinis* colonisation.^90^ Several investigators have attempted to target *Strep. mutans* by various means^91–93^ and have evaluated the effects of numerous reagents on *Strep. mutans* biofilms.^94^

With respect to the use of lantibiotics against oral biofilms, Tong and co-workers examined the anti-biofilm activities of nisin, independently and in combination with free amino acids in targeting *Strep. mutans* biofilms.^95^ The results of crystal violet biofilm assays indicated that mixtures of either the l or d-enantiomers of Glu, Asp or Cys in combination with nisin could ameliorate the potency of nisin against biofilms of *Strep. mutans*.^95^ Indeed, free amino acids are an essential component of peptidoglycan and serve to regulate and disassemble biofilms.^95^ The potency of nisin in inhibiting insoluble glucan-biofilm synthesis by *Strep. mutans* 10449 was evaluated in another relatively recent study.^96^ It was found that 100 pmol of pure nisin prevented *Strep. mutans* 10449 from forming an insoluble glucan biofilm, after 1 h of cultivation. In contrast, a four-fold lower concentration of encapsulated nisin in liposomes was needed to have the same inhibitory effect on biofilm formation, after 2 h of cultivation. This is most likely due to the slower release of the peptide from nisin-liposomes, leading to prolonged anti-biofilm activity, compared to that of non-encapsulated naked nisin, whose inhibitory activity was relatively short-lived. Encouragingly, 30 pmol nisin in an encapsulated liposome form was effective at preventing biofilm formation by strain 10449 for a period of 6 h, in contrast to 30 pmol of nisin that was not encapsulated, which failed to maintain inhibitory activity for the same duration of time. Significantly, the results from the study highlight the effectiveness of encapsulated nisin in liposomes for a prolonged release of the peptide.^96^ An interesting study by Corbin et al. investigated the penetrative capabilities of nisin with a view to targeting dental biofilms. The application of nisin elicited a loss of green fluorescence, which was most notable along the cell cluster edge, relative to the centre of the cluster. However, nisin failed to cause complete eradication of the biofilm.^97^ With regards to other lantibiotics targeting oral biofilms, one study evaluated the efficacy of the two-component lantibiotic, lacticin 3147, in inhibiting the formation of biofilms by *S. mutans* and it was found that 2× MIC (6.3 μM) of lacticin 3147 was effective at disrupting biofilm formation by *Strep. mutans*. However, the lantibiotic was not as effective against biofilms which were already 4 h old.^98^

With respect to other biofilm-forming organisms in the oral cavity, Tong and co-workers conducted a separate study in which they assessed the effect of adding nisin to MTAD (mixture of tetracycline, acid and doxycycline) and its anti-biofilm activity against *Enterococcus faecalis* isolates.^99^ In contrast to biofilms of the oral pathogen *Strep. mutans, E. faecalis* biofilms contain reduced levels of extracellular polysaccharides and higher quantities of extracellular DNA, resulting in lower impedance to penetration by antimicrobials.^100^ Importantly, it was found that nisin in combination with doxycycline successfully inhibited *E. faecalis* biofilms whereas MTAD on its own was ineffective against such biofilms. This successful combination could be harnessed as an antimicrobial/irrigant following root canal treatments to prevent post-operative *E. faecalis* infections. In this regard, it should be noted that aside from being implicated in the formation of oral biofilms, *E. faecalis* is an important nosocomial pathogen and has exhibited resistance to various classes of antibiotics. Indeed, *Enterococcus* species are implicated in endocarditis, catheter-related infections, urinary tract infections and infections associated with surgical wounds.^101–103^ Encouragingly, a recent study by Kajwadkar et al. revealed that nisin ZP, which is a naturally occurring variant of nisin A, independently and in combination with sodium hypochlorite was potent against *E. faecalis* biofilms and planktonic cells. Exposure of the biofilm to nisin ZP at concentrations >10 µg/ml for 10 mins were effective at decreasing the thickness and bio-volume of the biofilm, while combinations of nisin ZP with low concentrations of sodium hypochlorite were also found to be effective at reducing the biomass of the biofilm.^104^

*Actinomyces viscosus* is another member of the oral cavity and is frequently found as a biofilm in periodontal pockets.^87,105–107^ It has also been implicated in endocarditis, similar to *E. faecalis*.^108^ Balto et al. investigated the efficacy of the dental irrigant MTAD in combination with nisin on *A. viscosus* and *E. faecalis* biofilms on membrane filter discs. Unfortunately however, there was no difference in the viability of the *A. viscosus* and *E. faecalis* biofilms when treated with MTADN (MTAD with added nisin) and 5.25% sodium hypochlorite.^109^ Finally, a recent study demonstrated that the bacteriocin EntV produced by a strain of *E. faecalis*, prevents biofilm formation by the yeast *Candida albicans*.^110^ *C. albicans* biofilms are frequently associated with oral and vaginal thrush.^111,112^ The anti-biofilm activity of EntV is mediated via the disruption of hyphal formation, which is essential for *C. albicans* biofilm formation. Significantly, the study showed that the 68-amino acid peptide, EntV, prevented development of *C. albicans* biofilms (which were recalcitrant to several antifungals) on solid surfaces in various media conditions.^110^ Thus, this bacteriocin could have tremendous potential as a therapeutic agent for both oral and vaginal thrush.

A summary of studies involving lantibiotics utilised in an effort to target biofilms is found in Table 1.

**Table 1:** Activity of the lantibiotic group of bacteriocins against biofilms

Bacteriocin(s)	Biofilm former	Effects	Reference
Nisin in combination with antibiotics	MRSA	Nisin-antibiotic combinations prevented biofilm formation	^12^
Nisin with ciprofloxacin/daptomycin	MRSA	Synergy between nisin and ciprofloxacin/daptomycin against biofilm	^45^
Nisin, lacticin Q, nukacin ISK-1	MRSA	Nisin and lacticin Q potent against biofilm, causing pore formation, efflux of ATP from biofilm. No anti-biofilm activity for nukacin ISK-1	^46^
Nisin, bovicin HC5	*S. aureus*	Reduced adhesion to polystyrene. Reduced expression of genes involved in biofilm formation	^51^
Nisin and lysozyme	25 *S. aureus* strains	1× MIC nisin prevented biofilm formation	^52^
Gallidermin	*S. aureus, S. epidermidis*	Prevention of biofilm formation. Persister cells survived	^53^
Nisin	*S. epidermidis*	Loss of green fluorescence from biofilm, loss of viability and membrane integrity	^28^
Nisin I4V	*S. pseudintermedius*	I4V inhibited formation and reduced biomass of biofilms	^31^
Nisin I4V	*S. pseudintermedius*	I4V potent against DSM21284 biofilms	^32^
Nisin M21A with citric acid or cinnamaldehyde	*L. monocytogenes* F2635	Nisin combined with essential oils effective against biofilm	^68^
Nisin	*L. monocytogenes* 4032	Mature biofilms on stainless steel and polypropylene recalcitrant to nisin	^69^
Nisin	*S. aureus, L. monocytogenes, Salmonella enteritidis*	4000 IU/ml particularly effective against *Salmonella enteritidis* and *L. monocytogenes* biofilm formation	^71^
Nisin at low pH with high hydrostatic pressure (HHP)	*L. monocytogenes*	Nisin at low pH combined with HHP effective against biofilms	^72^
Nisin and polymyxin	*P. aeruginosa*	Inhibition of biofilm formation. Dose of polymyxin required lowered	^73^
Nisin in combination with Glu, Asp, Cys	*Strep. mutans*	Improved potency against *Strep. mutans* biofilms with nisin-amino acid combinations	^95^
Nisin	*Strep. mutans* 10449	Inhibition of glucan biofilm synthesis. Encapsulated nisin most effective	^96^
Nisin	Oral biofilm	Loss of green fluorescence across biofilm cell clusters	^97^
Lacticin 3147	*Strep. mutans*	2× MIC disrupted formation of biofilms. Ineffective against 4-h-old biofilms	^98^
Nisin and MTAD	*E. faecalis*	Nisin and doxycycline inhibited *E. faecalis* biofilms	^99^
NisinZP and sodium hypochlorite	*E. faecalis*	Thickness and bio-volume of biofilm decreased	^104^
Nisin and MTAD	*E. faecalis, A. viscosus*	No effect on biofilms	^109^
Subtilomycin, lichenicidin and nisinZ	*L. monocytogenes*	Prevention of biofilm formation	^70^
EntV	*C. albicans*	Prevention of *C. albicans* biofilm formation	^110^

## Anti-biofilm activity of other groups of bacteriocins

While the majority of studies with bacteriocins used to target biofilms have used lantibiotics, other groups of bacteriocins have also been investigated. Recently, Chopra et al. reported the discovery of a novel bacteriocin, sonorensin, which possessed potent anti-biofilm activity. Sonorensin belongs to the heterocycloanthracin subclass of bacteriocins.^113,114^ This novel bacteriocin exhibited potent activity against *S. aureus* biofilms and it was unveiled that the inhibition of biofilm growth could be ascribed to increased membrane permeability.^113^ The antimicrobial effects of enterocin AS-48 (a class IIc circular bacteriocin) both independently and in combination with several biocides against three MRSA and three methicillin-sensitive *S. aureus* (MSSA) strains was also assessed in a separate study by Caballero Gómez and co-workers.^115–117^ Caballero Gómez and co-workers found that the anti-biofilm activity of the biocides triclosan, benzalkonium chloride and polyhexamethylene guanidium chloride were highly effective in combination with 50 μg/ml of AS-48 against *S. aureus* biofilms in storage conditions.^115^ Another study demonstrated the efficacy of the class IIb bacteriocins enterocin DD93 and DD28 against MRSA biofilms. The enterocins were effective at inhibiting the formation of MRSA-1 biofilms on glass and SS surfaces when combined with the antibiotics kanamycin or erythromycin.^118^ Finally, a novel circular sactibiotic, hyicin 4244, produced by *Staphylococcus hyicus* 4244, has been shown to exhibit anti-biofilm activity against fourteen *Staphylococcus* strains which were the causative agents of either bovine or human mastitis.^119^ Significantly, the sactibiotic decreased the biofilm forming capacity of two of these strains as well as decreasing CFU (colony forming unit) counts. Furthermore, the sactibiotic elicited decreases in viability and growth of sessile cells in already established biofilms. Thus, not only was it effective at inhibiting biofilm formation, it was also able to traverse existing biofilms.

With respect to the inhibition of *L. monocytogenes* biofilms, Caballero Gómez et al. in another study found that enterocin AS-48 was effective at targeting planktonic cells but the organism was insensitive to 10 μg/ml AS-48 when cells were present in a sessile state.^120^ Thus, responses to the bacteriocins were markedly altered between *L. monocytogenes* cells existing in a planktonic state or sessile state. Interestingly, the authors found that in the sessile state, protein synthesis is prioritised in lieu of carbohydrate metabolism when cells are exposed to bacteriocins. Furthermore, stress response proteins such as GroEL and DnaK were overexpressed by biofilm cells upon exposure to the bacteriocin and some of these overexpressed proteins actually contribute to adhesion of the biofilm to surfaces,^120^ suggesting that exposure to sub-lethal concentrations of bacteriocins may in fact trigger biofilm formation in some instances. In contrast, an earlier study by the same group showed that AS-48 was effective when combined with several biocides against *L. monocytogenes* cells present in a sessile state.^121^ While high concentrations of AS-48 (50 μg/ml) on its own were required to inhibit *L. monocytogenes* biofilms attached to polystyrene plates, there was a marked improvement in the inactivation of biofilms when AS-48 was applied in combination with the biocides used in the study, with the exception of the sanitisers P3 topax 66 and P3 oxonia solutions. In addition, pre-treatment of polystyrene plates with 0.5–25 µg/ml AS-48 led to a reduction in adherence and consequent formation of *L. monocytogenes* biofilms.^121^ Finally, an interesting study recently reported the efficacy of the class II unmodified bacteriocin licheniocin 50.2 against biofilms of *L. monocytogenes* as well as against coagulase-negative *Staphylococcus* (CoNS) biofilms.^122^ CoNS are important clinically and amongst the most common CoNS pathogens are *S. epidermidis*, *S. haemolyticus*, *S. saprophyticus* and *S. lugdunensis*.^123–126^ Cirkovic and co-workers tested the efficacy of the two new bacteriocins against twelve *L. monocytogenes* and eight CoNS strains. It was noted that low concentrations of licheniocin 50.2 were effective at preventing CoNS biofilm formation while the crude extract from strain BGBUI-4 successfully prevented formation of biofilms by *L. monocytogenes*. In addition, 100 AU/ml and 200 AU/ml of the bacteriocins were effective at diminishing the biomass of 1 day-old *L. monocytogenes* and CoNS biofilms.^122^ Despite these promising outcomes, caution must be exercised when utilising crude extracts, as the apparent anti-biofilm activity observed might not solely be due to the presence of bacteriocins but additional compounds present in the extract as well.

With respect to targeting biofilms formed by Gram negatives, combinations of enterocin DD14, nisin and the antibiotic colistin proved successful at removing *E. coli* CIP54127 biofilms in one study.^127^ Enterocin DD14 is an unusual two-peptide class IIb bacteriocin devoid of a leader sequence.^128^ The *E. coli* strain CIP54127 has previously been reported to exhibit resistance to disinfectants including oxidising agents, phenolic derivatives and cationic or amphoteric surfactants, chiefly due to its biofilm-forming ability.^129^ Significantly, in addition to strain CIP54127, enterocin DD14, nisin and colistin combinations were also effective against biofilms of *E. coli* 184 (mcr-1^+^) and *E. coli* (mcr-1^−^), which are known to be resistant to colistin. Turovskiy et al. investigated the efficacy of the sactibiotic bacteriocin subtilosin, lauramide arginine ethyl ester (LAE) and Ɛ-poly-l-lysine against biofilms of the Gram variable pathogen *Gardnerella vaginalis*, using ATP viability, resazurin assays and plate counts.^130^ This pathogen is associated with recurrent bacterial vaginosis and its biofilm forming ability facilitates its colonisation and contributes to antibiotic resistance.^131,132^ While subtilosin exhibited antimicrobial activity against *G. vaginalis* biofilms, it was noteworthy that the study demonstrated that resazurin assays and ATP viability assays led to an underestimation of the bactericidal effect of certain antimicrobials, highlighting the limitations and the variability associated with certain viability assays.^130^ In a follow-up study, Algburi et al. determined that sub-inhibitory concentrations of subtilosin disrupts quorum sensing in *G. vaginalis* biofilms, as well as in biofilms formed by Gram negative bacteria.^133^ The peptide was shown to elicit a decrease in the production of autoinducer-2 (AI-2) within *G. vaginalis* biofilms and a similar reduction in the production of voilacein by a Gram negative reporter *Chromobacterium voilaceum* strain. Interestingly however, while sub-MIC levels of subtilosin were able to reduce the formation of *L. monocytogenes* biofilms, a concomitant decrease in AI-2 production was not observed, indicating that mechanisms other than quorum sensing inhibition may be responsible for subtilosin’s inhibitory activity against *L. monocytogenes* biofilms.^133^

Finally, it has been shown that the class IIb bacteriocin plantaricin A in the presence of *Lactobacillus sanfranciscensis* DPMMA174 and *Lactobacillus plantarum* DPPMA20 actually facilitated the formation of biofilms by *Lb. plantarum* DC400 (ref. ^134^). An increase in biofilm formation can perhaps be beneficial in some cases whereby a potential ‘probiotic’ biofilm former such as *Lb. plantarum* can confer protection against biofilms involving other potentially pathogenic strains.

## Application of bacteriocins onto abiotic surfaces with a view to inhibiting biofilms

Since established biofilms are notoriously difficult to control, perhaps a more logical approach is to apply bacteriocins onto certain abiotic surfaces, to prevent the formation of target biofilms on such susceptible surfaces. This strategy was utilised in a recent study by Al-Seraih et al., whereby the authors described the efficacy of enterocin B3A-B3B, as well as nisin, against *L. monocytogenes* biofilms.^135^ Enterocin B3A-B3B is a class IIb bacteriocin, highly similar in sequence to MR10A-MR10B. The enterocin disrupted the formation of *L. monocytogenes* 162 biofilms on SS. Furthermore, the study showed that application of 1 mg/ml or 16 mg/ml nisin onto the surface of SS elicited a 2-log cfu/ml reduction in cell numbers and consequently inhibited the formation of biofilms of *L. monocytogenes* 162 and 162R (nisin-resistant derivative), respectively. In addition, B3A-B3B combined with nisin proved to be effective as it led to a decrease in MIC needed to impede the growth of this pathogen in either biofilms or in a planktonic state.^135^ In another study, Nostro and co-workers assessed the efficacy of poly-ethylene-co-vinyl-acetate (EVA) films incorporated with nisin at inhibiting the biofilm-forming capabilities of *S. epidermidis* ATCC35984, *S. aureus* 815 and *L. monocytogenes* ATCC7644 (ref. ^136^). A combination of techniques including measurements of biofilm biomass, live/dead staining and fluorescence microscopy was used to assess anti-biofilm properties and it was established that EVA14 (nisin) films were more effective at diminishing the biofilm-forming abilities of *S. epidermidis*, and less effective against *S. aureus* and *L. monocytogenes* strains. These findings were validated by fluorescence microscopy which demonstrated markedly reduced biofilm formation on EVA14 films incorporated with nisin. In contrast, EVA28 (nisin) films failed to show any anti-biofilm activity.^136^ A separate study of this nature demonstrated that immobilised nisin with poly-ethylene glycol utilised as a linker, potentiated the anti-biofilm activity of multi-walled carbon nanotubes (MWNT).^137^ Indeed, this composite of nisin immobilised-MWNTs displayed a higher degree of antimicrobial activity against *S. aureus*, *Bacillus subtilis*, *P. aeruginosa* and *E. coli*. Significantly, this composite was effective at inhibiting the formation of biofilms either in suspension or on a deposited film. Encouragingly, the anti-biofilm properties of the nisin-MWNT deposit film was approximately 100-fold greater than MWNT deposit films without nisin.^137^ A similar separate study also evaluated the efficacy of a MWNT sheet which was coated with nisin (MWCNT), in inhibiting the formation of biofilms by *Bacillus anthracis*.^138^ *B. anthracis* is a notorious pathogen and is the causative agent of pulmonary, cutaneous and gastrointestinal anthrax. It has also been reported to be a strong biofilm former and such biofilms of *B. anthracis* exhibit resistance to several antibiotics.^139^ Significantly, Dong et al. noted that *B. anthracis* biofilm formation was inhibited by up to 94.6% with MWCNT sheets coated with nisin, relative to sheets which were uncoated, as it is likely that nisin prevented adhesion of the biofilm. The activity of nisin combined with 2, 3-dihydroxybenzoic acid (DHBA) in nanofibers and its ability to inhibit MRSA biofilm formation was also explored in a relatively recent study.^140^ The nanofibers which contained DHBA in combination with nisin were particularly effective, eliciting an 88% reduction in formation of MRSA biofilms after 24 h of exposure. Finally, in another study, nisin bound to SS surfaces was shown to decrease adhesion and consequently reduce the formation of biofilms of *Listeria ivanovii* on the surface.^141^

## Activity of uncharacterised bacteriocins and crude extracts against biofilms

There have been a number of studies conducted whereby a bacteriocinogenic strain was utilised in an effort to target biofilms. However, in many instances, the bacteriocin was not fully characterised at the time of the study. In some of these studies below, the uncharacterised bacteriocin was purified or semi-purified, prior to investigation, while in others, crude extracts of the uncharacterised bacteriocins or co-cultures of the bacteriocinogenic strain with the target biofilm-forming organism were utilised. It must be noted that the presence of other compounds in crude extracts of bacteriocins may be additional confounding factors, when evaluating anti-biofilm activity, as several of these compounds could possibly contribute to the apparent inhibitory activity.

With respect to investigations utilising purified or semi-purified bacteriocins, Ming et al. showed that bacteriocins produced by *Lb. plantarum*, which were purified by ammonium sulphate precipitation, caused an inhibition of biofilms of *Strep. sanguinis*.^142^ A separate study reported the discovery of a novel bacteriocin produced by the Gram negative, *Citrobacter freundii*, which possesses potent anti-biofilm activity. This novel bacteriocin was effective against both planktonic cells and biofilms of *Citrobacter* species, *E. coli* and *Klebsiella pneumoniae*.^143^ *K. pneumoniae* is an important nosocomial opportunistic Gram negative pathogen, implicated in infections of the lung, as well as being able to form biofilms on abiotic surfaces, such as urinary catheters.^144,145^ The purified bacteriocin from *C. freundii*, once expressed in a heterologous *E. coli* host, also possessed potent activity against biofilms of *K. pneumoniae*, *E. coli* and *Citrobacter* species.^143^ Biofilms of the Gram negative pathogen *Serratia marcescens* were also targeted using purified and partially-purified bacteriocins produced by *Lb. plantarum* ATCC 8014 and *Lactobacillus acidophilus* ATCC4356 respectively.^146^ *Ser. marcescens* has a propensity to form strong biofilms, largely determined by the availability of nutrients in the environment. It has been associated with negative transfusion reactions caused by contaminated platelet concentrates and has also been implicated in urinary tract infections in hospitalised patients, exhibiting resistance to certain antibiotics.^147,148^

With regards to studies involving co-culture of bacteriocinogenic strains along with the target biofilm species, and/or involving crude extracts containing uncharacterised bacteriocins, one such study showed that the bacteriocin produced by *Lactobacillus sakei* CRL1862 was able to inhibit *L. monocytogenes* biofilm formation, and this anti-biofilm forming activity was more potent on polytetrafluoroethylene (PFTE) surfaces, relative to SS surfaces.^149^ In addition, co-culture of the bacteriocinogenic *Lb. sakei* strain along with the *L. monocytogenes* target elicited a decrease by 4.52 and 5.54-log on SS and PFTE surfaces respectively.^149^ Another study evaluated the efficacy of nisin as well as a bacteriocinogenic *Enterococcus faecium* strain on the ability of *L. monocytogenes* to form biofilms on SS coupons and in Brain Heart Infusion broth.^150^ Interestingly, co-culture of *L. monocytogenes* and the bacteriocinogenic *E. faecium* strain prevented *L. monocytogenes* adherence and biofilm formation for up to 2 days. The presence of nisin also led to a decrease in bacterial growth up to 4.6-log cfu/cm^2^ on SS coupons, in comparison to untreated *L. monocytogenes* cultures. Worryingly, however, nisin failed to kill the cells in the biofilm and a new biofilm layer was apparent in cultures exposed to nisin. Thus, while the presence of nisin led to a decrease in *L. monocytogenes* growth, the co-culture of *L. monocytogenes* with the bacteriocinogenic *E. faecium* strain was the most effective option for diminishing the formation of *L. monocytogenes* biofilms.^150^ A study by Camargo et al. investigated the efficacy of different bacteriocins against biofilms of *L. monocytogenes*. Interestingly, it was noted that the bacteriocin-containing cell free supernatants (CFS) from *Lactobacillus curvatus* ET31 effectively reduced the formation of *L. monocytogenes* biofilms (initial stages of biofilm formation). However, the CFS was ineffective after the biofilm had already formed. The CFS was also tested in combination with the chelating agent ethylene diaminetetraacetic acid (EDTA). While the bacteriocin and the EDTA independently were ineffective against *L. monocytogenes* biofilms which had already formed, the combination was effective at reducing the viability of established biofilms, whilst not fully eliminating the biofilms.^151^ The anti-biofilm activity of bacteriocins produced by *Lb. fermentum* 97 against biofilms of several enterotoxigenic enterobacteria, *S. epidermidis* and *C. albicans* has also been demonstrated.^152^ *C. albicans*, the causative agent of candidiasis, is a nosocomial fungal pathogen and has the ability to form relatively strong biofilms, particularly in the oral cavity.^153^ Indeed, candidiasis has been described as the most prevalent fungal infection in humans.^154^ Finally, Lin et al. found that *Strep. mutans* UA159 biofilm formation was inhibited through the production of bacteriocin-like polypeptides produced by *Lactobacillus* strains.^155^

A summary of studies involving other non-lantibiotic groups of bacteriocins and uncharacterised bacteriocins used to target biofilms is found in Table 2.

**Table 2:** Activity of other groups of bacteriocins and uncharacterised bacteriocins against biofilms

Bacteriocin(s)	Biofilm former	Effects	Reference
Sonorensin	*S. aureus*	Strong activity against *S. aureus* biofilms and inhibition of biofilm formation	^113^
Enterocin AS-48 with benzalkonium chloride, polyhexamethylene guanidium chloride and triclosan	MRSA and MSSA	50 µg/ml enterocin with biocides effective against MRSA biofilms	^115^
Enterocin DD93, DD28 with erythromycin or kanamycin	MRSA	Combinations prevented biofilm formation	^118^
Enterocin AS-48	*L. monocytogenes*	10 µg/ml AS-48 ineffective against sessile cells	^120^
Enterocin AS-48 with biocides	*L. monocytogenes*	50 µg/ml AS-48 needed to inhibit biofilm forming on polystyrene. More effective in combination with biocides	^121^
Licheniocin 50.2	*L. monocytogenes*, coagulase negative-staphylococci	Prevented formation of biofilms	^122^
Nisin, enterocin DD14, colistin combination	*E. coli* CIP54127, *E. coli* 184 (mcr-1^+^) and *E. coli* (mcr-1^−^)	Removal of biofilm	^127^
Subtilosin, LAE, ε-poly-l-lysine	*G. vaginalis*	Effective against biofilm	^130^
Hyicin 4244	*Staphylococcus* strains	Reduced formation of biofilms. Reduced viability and growth of sessile cells	^119^
Plantaricin A	*Lb. plantarum* DC400	Increased biofilm formation when plantaricin A was combined with *Lb. sanfranciscensis* and *Lb. plantarum* strains	^134^
Various bacteriocins with EDTA. *Lb. curvatus* ET31 CFS.	*L. monocytogenes*	CFS potent at preventing biofilm but ineffective against formed biofilms.	^151^
Bacteriocin from *Lb. sakei* CRL1862	*L. monocytogenes*	Biofilms targeted on PFTE, SS surface	^149^
Nisin and bacteriocin from *E. faecium*	*L. monocytogenes*	Co-culture prevented biofilm formation	^150^
Bacteriocin from *Lb. fermentum 97*	*S. epidermidis*, *C. albicans*, enterotoxigenic enterobacteria	Effective against *S. epidermidis* biofilms	^152^
*Lactobacillus* strains producing bacteriocins	*Strep. mutans*	Prevention of biofilm formation	^155^
*Lb. plantarum* producing bacteriocins	*Strep. sanguinis*	Anti-biofilm activity against *Strep. sanguinis*	^142^
Bacteriocin from *Citrobacter freundii*	*Citrobacter, K. pneumoniae, E. coli*	Potent against biofilms	^143^
Bacteriocins from *Lb. plantarum* ATCC8014 and *Lb. acidophilus* ATCC4356	*Ser. marcescens*	Biofilm inhibited	^146^

## Conclusions

Due to the dearth of novel antibiotics in recent years, and the on-going crisis of antimicrobial resistance, bacteriocins may yet prove to be invaluable in clinical settings against biofilm producers. It is noteworthy that thus far, only the derivative of the lantibiotic deoxyactagardine B, NVB302, has undergone clinical trials and in general bacteriocins have failed to gain traction as alternative/adjunct therapeutic options in the clinic.^156,157^ Despite the paucity of clinical trials conducted with bacteriocins however, some key in vivo studies have assessed the efficacy of bacteriocin producers as potential probiotics. One example of a bacteriocin producer which could be harnessed as a probiotic is *Lactobacillus salivarius* UCC118, which produces the bacteriocin Abp118. A murine trial showed the ability of this bacteriocin producer to successfully colonise mice infected with *L. monocytogenes*.^158^ In a follow-up study, it was discovered that bacteriocin gene expression was up-regulated when UCC118 adhered to epithelial cells, most likely being mediated by an induction peptide present in high concentrations.^159^ Other such examples include a study which showed that colicin-producing *E. coli* strains survived for a longer duration of time in the large intestine of mice treated with streptomycin, compared to non-colicin producing derivatives, indicating that bacteriocin production could be considered a probiotic trait.^160^ For comprehensive reviews highlighting the potential role of bacteriocin production as a probiotic trait, the reader is referred to Hegarty et al.^161^ and Dobson et al.^162^

With respect to evaluating the effectiveness of antimicrobials against biofilms, several different types of assays including a variety of fluorescence microscopy techniques, cell viability and metabolic activity-based assays, and microtitre-based dye staining methods to measure biofilm biomass, are used to measure anti-biofilm activity (for a comprehensive review, see Azeredo et al.).^163^ While some of these assays quantify the removal of the pre-formed established biofilm biomass, other assays merely quantify the reduction in the ability to form biofilms due to the activity of the antimicrobial in question against planktonic cells. Perhaps a combination of some of these techniques is warranted to gain comprehensive insights into the inhibitory activity of the anti-biofilm agents being investigated, as each of these assays presents its own advantages as well as limitations.

A number of studies have identified effective bacteriocin–antimicrobial combinations with regards to targeting biofilms comprised of pathogens. It is conceivable that effective combinations of this nature will also attenuate the likelihood of development of antimicrobial resistance. Amongst the key advantages of bacteriocins is that oftentimes, they can be more potent than antibiotics in targeting planktonic cells and/or biofilms. Furthermore, the bioengineering of lantibiotics and indeed other groups of bacteriocins can unleash a plethora of derivatives with ameliorated bioactivity and other favourable physicochemical properties, many of which may be useful against biofilms. Indeed, advances in molecular biology techniques and high throughput facilities have led to the creation of a greater number of banks of bioengineered bacteriocins in the last decade or so.^19,20,24^ Critically, it has also been shown that the most thoroughly studied bacteriocin, nisin, is not broken down or trapped in the matrix within a biofilm, thus facilitating its further penetration into the inner depths of the biofilm.^28^ It must be noted however, that the lantibiotic may well be cleaved due to the presence of proteases in vivo, especially if administered orally. Nonetheless, it was particularly noteworthy from the study by Davison et al. that although the molecular weight of nisin is approximately ten-fold higher than quaternary ammonium compounds (QAC), the bacteriocin was able to penetrate through to the depths of the biofilm cell cluster quicker than QACs. It is plausible that synthetic antimicrobials which possess enhanced diffusion properties with a structure similar to nisin could also be developed in order to traverse the stratified layers of biofilms. Furthermore, it may also be possible to bioengineer other groups of bacteriocins to improve their diffusion properties with a view to penetrating biofilms. Indeed, optimisation of the aqueous diffusion coefficient of a bacteriocin peptide is likely to be critical for effective transit across the strata of the biofilm.^28^

However, perhaps a better option to circumvent the antimicrobial resistance that is inherent in biofilms is to seek synergistic combinations of bacteriocins with other antimicrobials that possess proven anti-biofilm properties. Any such synergistic combinations are likely to retard the development of resistance to either of the antimicrobials used in combination. Furthermore, effective synergistic combinations between bacteriocins and antibiotics are likely to reduce the concentrations of expensive antibiotics required to target biofilms. While this can be advantageous in certain instances due to the fact that some antibiotics such as the polymyxins can trigger undesired side effects such as nephrotoxicity at higher concentrations in clinical situations,^164^ it must be highlighted that the presence of sub-inhibitory concentrations of antibiotics may actually trigger the development of antibiotic resistance, as has been described previously.^165–168^

**Fig. 3:**
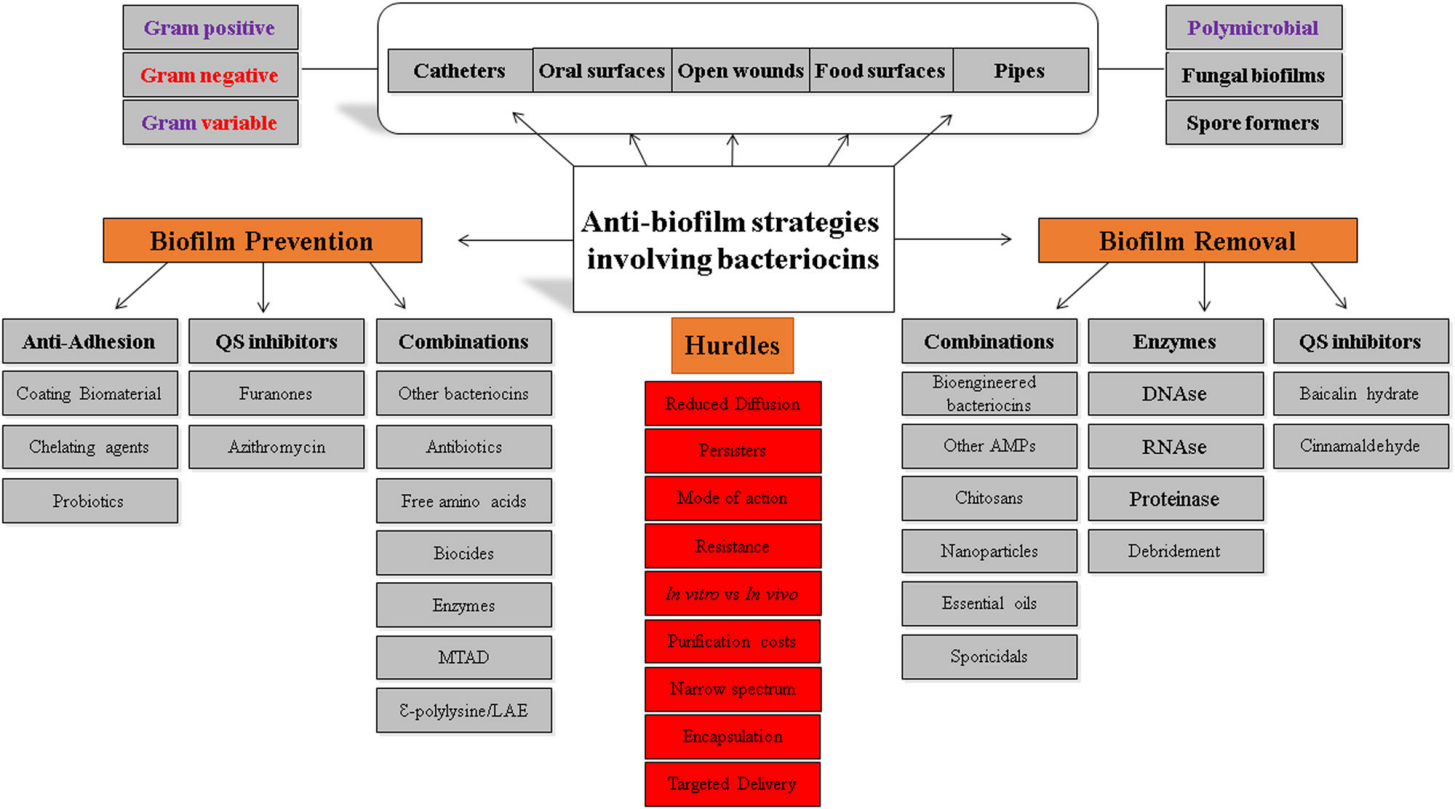
Strategy map of anti-biofilm activity of bacteriocins: Biofilms can be formed by a variety of organisms on both biotic and abiotic surfaces, including catheters, oral surfaces, wounds, food and stainless steel pipes. Bacteriocins could be utilised independently and in combination with other antimicrobials, quorum sensing inhibitors, biofilm degrading enzymes to inhibit biofilm formation and/or eradicate existing biofilms. However, a number of bottlenecks and knowledge gaps must be addressed for this strategy to be successful. QS quorum sensing, MTAD mixture of tetracycline, acid and doxycycline, LAE lauramide arginine ethyl ester, AMP antimicrobial peptide

Despite numerous potential advantages associated with using bacteriocins, they are by no means panaceas to target recalcitrant biofilms and several bottlenecks do exist with bacteriocins targeting biofilms as well. Biofilms are notoriously difficult to remove and high concentrations of antimicrobials are required to inhibit them once they’re formed. As is the case with many antimicrobials, bacteriocins in general seem to be more effective at inhibiting the formation of biofilms but exhibit reduced efficacy at targeting biofilms which are already formed. Another fundamental bottleneck with bacteriocins is the relatively low production of peptide(s) made by natural producers and the potential expense associated with purification of any such low-yielding peptide(s). However, advances in screening as well as optimisation of purification technologies have the potential to somewhat curtail these limitations. It is also possible that the production of a bacteriocin in a heterologous host can increase the yield of the peptide(s) produced, thereby reducing the costs involved in purification. Due to the proteinaceous nature of bacteriocins, the secretion of proteases by biofilms also has the potential to render bacteriocins and indeed other antimicrobial peptides ineffective and one study has already described proteases released by *P. aeruginosa* biofilms upon exposure to the antibiotic ciprofloxacin.^169^ However, it may be possible to mitigate this potential proteolysis via bioengineering of protease-resistant derivatives of bacteriocins. Other obstacles include a lack of detailed insights into the mode of action of several of these bacteriocins against biofilms. Further studies such as those conducted for gallidermin, which showed transcriptional changes of *atl* and *ic* genes in *S. aureus* biofilms,^53^ and nisin and lacticin Q, which were shown to elicit ATP efflux from cells in a biofilm,^46^ could prove invaluable in this regard. Furthermore, there is a lack of data concerning the propensity for bacteriocin-resistance development in cells within a biofilm. The narrow-spectrum nature of certain bacteriocins would render them ineffective against polymicrobial biofilm communities, in which the specific composition of the biofilm may be largely unknown. Other potential obstacles include elucidating the optimum dose of the bacteriocin required to eradicate biofilms, as doses required in therapeutic cases may not necessarily correlate with in vitro findings. Finally, it may be the case that penetration of certain high molecular weight bacteriocins, compared to antibiotic counterparts, across the strata of the biofilm may be the rate-limiting factor governing the potential success of bacteriocins in a biofilm setting. A combination of innovative strategies to prevent/retard the formation of a biofilm are warranted, examples of which include inhibiting quorum sensing and communication within a biofilm, in combination with potent bacteriocins, to fully combat a biofilm (Fig. 3).

Perhaps the most common, yet largely under-appreciated biofilm that exists in the human body is dental plaque, which consists of bacteria embedded in a proteinaceous and polysaccharide-rich matrix and is the causative agent of dental caries and periodontal disease.^88,170,171^ It is interesting to note in this regard that dental plaque is a very complex biofilm consisting of several streptococci and many of these streptococci actually produce bacteriocins called mutacins in situ, which can control the composition of the species that exist in the biofilm, giving certain strains a competitive advantage over others in the biofilm.^172–174^ Indeed, a complex microbiome exists within an oral biofilm and the precise composition of this microbial community is likely to determine the caries-causing potential of such a biofilm. Depending on the nature and target specificity of any mutacins produced in situ, it is possible that the cariogenic potential of dental plaque can either be attenuated or in the worst-case scenario, even enhanced by targeting strains associated with good oral health. Nonetheless, a ‘probiotic’ *Streptococcus* strain producing mutacins or other bacteriocins could potentially be harnessed to inhibit other biofilm-forming and caries-causing *S. mutans* strains. Despite decades of research seeking antimicrobials which are effective at targeting dental plaque however, it is widely accepted that the most efficient and cost-effective means to eradicate this particular biofilm is via simplistic mechanical debridement, by regularly brushing, flossing and scaling teeth.

Overall, minor differences in the 3D-structure of the polymeric matrix and/or alterations in the physiology of bacteria that exist in a biofilm compared to a planktonic state are likely to contribute to variations in sensitivity to bacteriocins and indeed other antimicrobials. Thus, the ideal ratio of a bacteriocin mixed with another stressor may play a key role in optimising the synergistic effects that can be obtained against biofilms or planktonic cells. Furthermore, strategies which involve combining bacteriocins or bioengineered derivatives thereof, with other antimicrobials such as thiazolidinone derivatives, diterpenoids, epigallocatechin, baicalin hydrate as well as other enzymes/agents that interfere with molecular pathways involved in the formation of biofilms could prove to be successful.^175^ Since several of these target biofilms via non-microbicidal means, it is plausible that combinations with a bacteriocin can be highly efficacious both in terms of inhibiting the biofilm, as well as attenuating the likelihood of development of resistance to the bacteriocin.^176^ Greater insights into the precise structure-function properties of a biofilm matrix as well as the mode of action of several of these bacteriocins with anti-biofilm activity will have to be elucidated. Such insights are likely to facilitate the successful treatment and eradication of biofilms. Thus, while bacteriocins have several benefits as well as shortcomings, this group of ribosomally synthesised antimicrobials may yet prove to be critical players in the fight against biofilms, particularly when utilised in combination with other existing anti-biofilm agents.
